# Preventing HIV in women in Africa

**DOI:** 10.1038/s41591-025-03535-8

**Published:** 2025-02-13

**Authors:** Frances M. Cowan, Maryam Shahmanesh, Paul A. Revill, Joanna Busza, Euphemia L. Sibanda, Sungai T. Chabata, Natsayi Chimbindi, Tamara Choola, Owen Mugurungi, James R. Hargreaves, Andrew N. Phillips

**Affiliations:** 1 Liverpool School of Tropical Medicine, Liverpool, UK; 2 Centre for Sexual Health and HIV AIDS Research (CeSHHAR) Zimbabwe, Harare, Zimbabwe; 3 Institute for Global Health, University College London, London, UK; 4 Africa Health Research Institute, Durban, South Africa; 5 University of KwaZulu-Natal, Durban, South Africa; 6 University of York, York, UK; 7 London School of Hygiene and Tropical Medicine, London, UK; 8 AFROCAB Treatment Access Partnership (AFROCAB), Lusaka, Zambia; 9 Ministry of Health and Child Care Zimbabwe, Harare, Zimbabwe

## Abstract

HIV incidence is declining globally, but around half of all new infections are in sub-Saharan Africa—where adolescent girls and young women bear a disproportionate burden of new infections. Those who sell sex are at particularly high risk. Despite declining incidence rates and availability of effective biomedical prevention tools, we are not on track, globally or in Africa, to achieve UNAIDS 2025 prevention targets. For those at risk, interventions that strengthen their motivation, capabilities and access to all available HIV prevention technologies are critical—for adolescent girls and women in particular, but also for epidemic control more broadly. Exciting possibilities for scaling up new and highly effective prevention technologies are close, but delivery, implementation and financing models need to be developed and urgently evaluated, in partnership with communities, or these opportunities may be lost. Here, we discuss the evolving landscape of biomedical prevention technologies for women in Africa, their implementation and financing, as well as priorities for HIV prevention research in this setting.

Between 2010 and 2023, global HIV incidence decreased by 39% to 1.3 million new infections per annum^1^, far short of UNAIDS’ target of 370,000 by 2025. Currently, just under half of all new infections are in sub-Saharan Africa (SSA). As elsewhere, changes in incidence within SSA vary by region, with slower declines in western and Central Africa (46%) compared to eastern and southern Africa (59%). The absolute number of new infections, however, remains over twice as high in eastern and southern Africa (450,000) than western and Central Africa (190,000)^2,3^.

Across the African continent, adolescent girls and young women carry a disproportionate burden of new HIV infections^4^. This is in part due to changing demographics; 40% of Africans are aged <15 years^5^. Adolescent girls and women who exchange sex for gifts or money are at particularly high risk^6^. Women at high risk of HIV acquisition also have high prevalence of other sexually transmitted infections (STIs; including chlamydia, gonorrhea, trichomonas and herpes)^7^ as well as of unintended pregnancy, which is often a greater concern than HIV^8^. Enabling all women at high risk of HIV acquisition to protect themselves is crucial for the individuals themselves and for epidemic control more broadly.

The global community is not on track to achieve UNAIDS 2025 prevention targets despite availability of highly effective prevention tools including condoms^9^, pre-exposure prophylaxis (PrEP), post-exposure prophylaxis (PEP) and tools to enable those with HIV to be rapidly diagnosed and started on antiretroviral therapy. The latter includes cheap point-of-care HIV lateral flow tests that can be provider-delivered or self-delivered^10^, rapid treatment initiation with highly effective drugs that lead to viral suppression (when adhered to) in people living with HIV^11^, which substantially reduces transmissibility^12^.

The Global HIV Prevention Coalition (https://hivpreventioncoalition.unaids.org/en/) has developed a ten-step road map^13^ to support countries to accelerate progress toward effective prevention coverage. This includes using data to identify gaps and monitor program coverage and quality in real time, focusing on ‘precision prevention’, that is, tailoring services and support to individuals or groups, reducing access barriers and creating an enabling environment. The HIV prevention cascade can be used as a framework to guide effective coverage^14,15^ and can help to identify key reasons for gaps at each step of prevention (Box 1). Such guiding frameworks and models can help to inform prevention strategies but need to be informed by, and adapted to, local contexts.

In this Review, we summarize the current epidemiological trends and the effectiveness of ongoing prevention strategies and their implementation among women in Africa. We also discuss how the prevention landscape might evolve with the introduction of additional prevention technologies and suggest priorities for future research.

## Populations at high risk of HIV acquisition

UNAIDS estimates that 25% of all new infections that occurred across SSA in 2022 were among key populations and their sexual partners^6,16^. UNAIDS defines key populations as those people who are at higher risk of HIV and other health issues due to their behaviors; this includes female sex workers^17,18^. HIV incidence among female sex workers is nine times higher than among all women, with this difference over 20 times greater in western and Central Africa and five times greater in eastern and southern Africa^19^. Young women who sell sex are especially vulnerable, because they experience relationship power imbalances due to their age as well as their gender. All women who sell sex do so against a background of intense stigma and discrimination, exacerbated by criminalization of sex work^20^. These challenges are likely to increase as a result of the recent erosion of sexual and reproductive rights in parts of Africa, the impact of climate change and the rise in conflicts, all of which negatively affect engagement with prevention services^10^.

As HIV epidemics become better controlled across the continent, the relative importance of these ‘key’ populations is likely to increase. In this scenario, failure to keep sex workers healthy could result in in repeated outbreaks of HIV as a result of direct or indirect transmission through sex work^21^. Modeling suggests that increasing the intensity of HIV prevention programs for female sex workers in SSA would offer cost-effective and beneficial impacts on population incidence^22^.

On the other hand, half of all new infections in SSA are in the general population, and while many of the risk factors for incident infection are known (for example, place of residence, young age, STIs, non-cohabiting with sex partners, selling sex, violence), general population prevention services have struggled to reach those at highest risk who are not easily identifiable^23,24^. Importantly, not all adolescent girls and women are equally at risk. In an analysis of population-representative surveys conducted across Africa between 1985 and 2020, the proportion of women reporting ever having sex before age 18 varied by region from under 25% to over 77% (highest in West and Central Africa) and the median age of sexual debut was above 18 in both eastern and southern Africa—the regions with highest rate of new infections (although there is considerable within-region variation)^25^. Without doubt, new approaches are required. Evidence from the Isisekelo Sempilo trial in rural South Africa showed that offering home-based sampling for STI testing and integrated sexual and reproductive health combined with HIV mobile services, increased uptake of HIV prevention by 60%^26^. Other models offer PrEP and family planning in the same facilities but from different providers^27,28^. However, these integrated models have not been scaled up for primary HIV prevention delivery in SSA^29^.

**Fig. 1:**
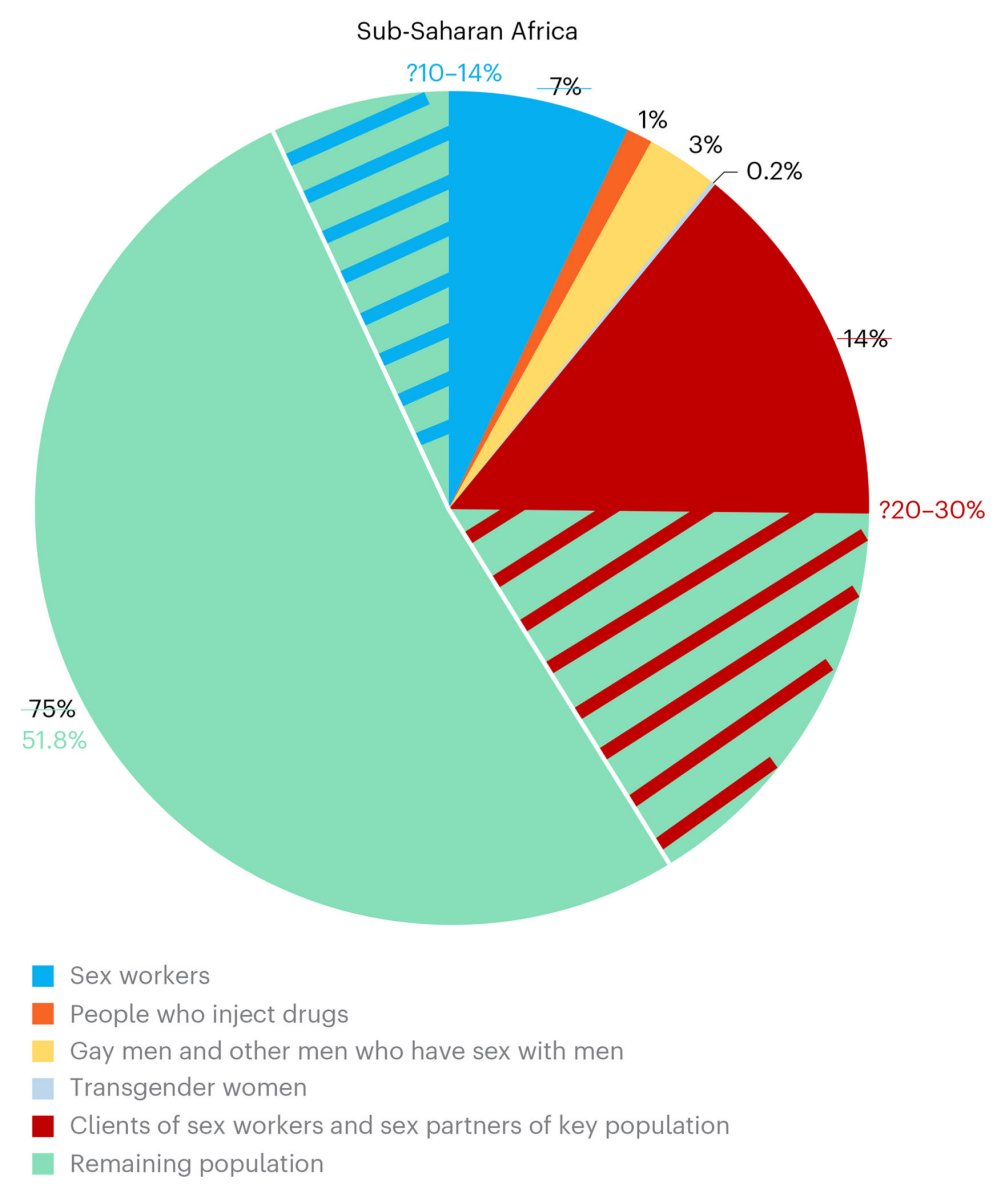
Women and men on the edge of sex work. The distribution of new HIV infections across key population groups in SSA in 2022 (data from UNAIDS Global AIDS Update 2024)^1^. Women who sell sex but do not identify as ‘sex workers’ (for example, when they first start selling sex, they sell sex only sporadically or are transitioning into and out of sex work) may make up a substantial proportion of infections, so that this group may be larger than the data suggest (segment with blue diagonal lines). Their male sex partners may also may make up a substantial proportion of new infections in men (segment with red diagonal lines).

Some of those at highest risk are likely individuals who sell sex but do not identify as sex workers (Fig. 1). A review from the mid-2000s found that when women in Africa were asked (in demographic and health surveys) about exchanging sex for gifts or money in the past 12 months, roughly twice as many women responded affirmatively compared to the number reporting being sex workers^30^. In Zimbabwe, young women who report selling sex but do not identify as sex workers tend to have fewer sex partners than those who do identify as sex workers, but their rate of HIV acquisition is just as high^31^. Similarly, in rural KwaZulu-Natal, South Africa, incidence of HIV infections among young women who sell sex (17% of young women overall) was 8% per annum, three times that of young women who did not report selling sex^32^. A comprehensive review of over 300 social science studies exploring transactional sex in SSA observed three overlapping motivations; sex for basic needs, sex for improved social status and sex and material expression of love. Crucially, the associated risks and appropriate mitigation approaches differ between these^33^.

Expanding tailored services for key populations—for example, by introducing peer-led social and sexual network approaches—may reach this population of women who transact sex but who, as a group, are less well characterized than self-identifying female sex workers. By contrast, most current strategies seek to identify and reach high-risk individuals from within ‘general population’ platforms (which include many people at very low or no risk). We argue that research to explore the relative merits (and cost-effectiveness) of both these approaches is needed. In addition, working cooperatively with these groups of women may help researchers to better understand who needs which services and where, and to identify optimal ways to package services, such as STI testing and sexual and reproductive health.

Other populations with high rates of HIV acquisition in SSA include tertiary education students, who report condomless sex, multiple partnerships, transactional sex and sex while intoxicated^34,35^. Pregnant and lactating adolescent girls and young women are another group with high HIV incidence, with new HIV infections associated with higher rates of mother-to-child transmission than preexisting HIV infections^36^. This group is relatively easy to identify and access through maternal and child health clinics. Although PrEP is recommended for this population^37^, and research shows its provision to be feasible and safe^38^, PrEP has not been adequately integrated into antenatal care^39–41^.

## Primary biomedical prevention strategies for women

PrEP and PEP agents have been rolled out to varying degrees (and with varying levels of success) in African countries, while recent advances in drug development are generating longer-lasting and more-effective options. In practice, however, prevention choices for women in Africa are often limited to condoms and daily oral PrEP. Below, we discuss the effectiveness, cost-effectiveness and availability of PrEP and PEP options for women in Africa. Other strategies such as preventive vaccines and broadly neutralizing antibodies are also being developed and tested, but these are not currently in a position to have real-world impact, so they are not discussed here.

### PrEP

Since 2015, the World Health Organization (WHO) has recommended daily oral PrEP^42^ for people at substantial risk of HIV (defined as HIV incidence greater than 3% per annum in their population group). More recently, longer-acting PrEP options—such as the monthly dapivirine vaginal ring (DVR) and injectable long-acting cabotegravir (CAB-LA; administered every 2 months)^43–46^—have been recommended and pre-qualified by the WHO, and are being scaled up to varying extents across Africa. Globally, 76 countries have national PrEP guidelines in place, with the global oral PrEP target set at 10 million individuals initiated by 2025. As of July 2024, only 7.5 million people had initiated oral PrEP globally^47^. According to the Global PrEP Tracker (https://data.prepwatch.org/), scale-up in Africa started in 2016 and by mid-2024, 3.7 million people in eastern and southern Africa and 706,000 in western and Central Africa had started oral PrEP. In South Africa, where more than 5.25 million people are eligible, only 1.35 million (<26%) had received a prescription by June 2024; effective use is likely lower^48^. PrEP rollout is crucial to achieving targets for HIV prevention, but unless careful consideration is given to implementation, for example, through service integration and task shifting, PrEP rollout could overburden already weak health systems^49^.

**Fig. 2:**
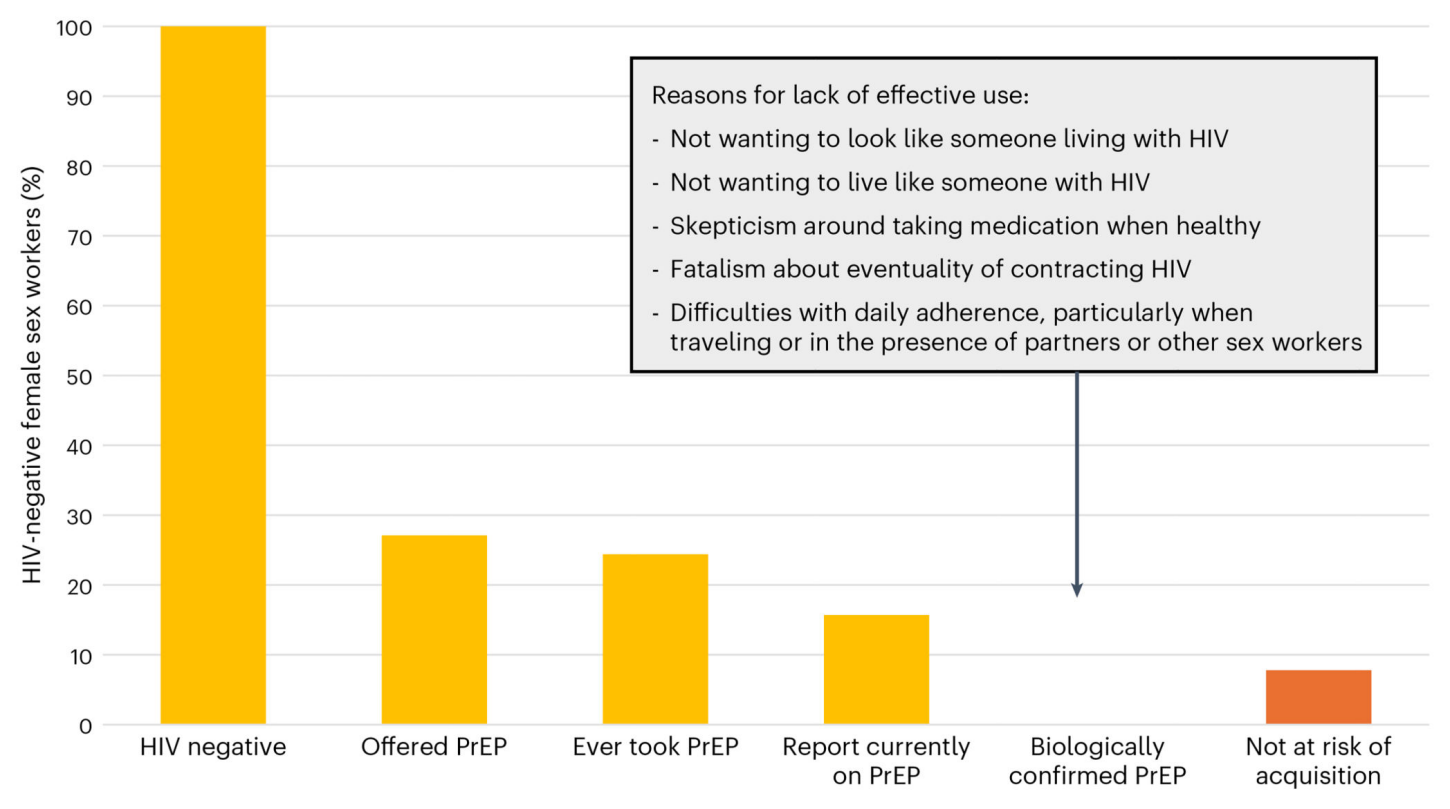
A PrEP cascade. The percentage of female sex workers who are HIV-negative (seronegative)–recruited to the endline survey of the AMETHIST trial^53,101^–who engaged with each step of the cascade, highlighting the substantial challenges to successful PrEP rollout.

Qualitative and quantitative data suggest a number of barriers to oral PrEP uptake and effective continuation^50,51^ (Box 2). Even among self-reported PrEP users, biomarkers often show low rates of actual protection—for example, among a sample of adolescent girls and young women who were regular PrEP program attendees in Kenya, fewer than 5% had protective plasma levels of tenofovir disoproxil fumarate (TDF; daily regimen). And among 578 female sex workers in Zimbabwe who reported current PrEP use in an end-of-trial survey (the AMETHIST trial), only 2 had TDF levels indicative of adherence at four or more doses per week^52–54^ (Fig. 2). There is some evidence that discussing TDF levels with users can reinforce adherence. The randomized, controlled HPTN 082 trial did not show any impact of this approach, although it had only modest power to detect such differences^55^; while the INSIGHT cohort^56^, an uncontrolled study that recruited over 3,000 adolescent girls and young women from several countries in Africa, showed high oral PrEP uptake (>95%) and continuation (>85% at 6 months) with this kind of communication. In this study, two-thirds of those tested at 6 months had detectable TDF; self-reported adherence aligned very well with positive results from the TDF test (in this case, a urine test) and over half the girls interviewed at 6 months reported that their urine TDF test result motivated them to take PrEP.

The DVR offers an alternative strategy—a female-controlled HIV prevention method for women who do not want to take tablets or have injections. It consists of a flexible silicone ring that is inserted in the vagina and slowly releases the antiviral dapivirine over the course of one month. Very little drug is absorbed systemically. In trials, the ring reduced risk of HIV acquisition by 35%, or as much as 45% with optimal adherence^45,46^. It has been conditionally recommended by the WHO since 2021 but is currently only licensed for use in a few African countries, and is not available outside implementation studies. A 3-month vaginal ring is currently being evaluated. Aphase 1 trial (https://popcouncil.org/project/the-three-month-dapivirine-vaginal-ring-for-hiv-prevention/) to investigate the relative bioavailability of dapivirine with the monthly versus 3-monthly DVR found that the latter delivers dapivirine at higher levels than the former.

Injectable PrEP is another long-lasting option that negates the need for taking pills daily. It is highly effective and widely preferred (over daily PrEP) in values and preferences studies^57^. In 2021 and 2022, large trials of injectable CAB-LA (given every 2 months) demonstrated its superiority compared to daily oral TDF in both men^43^ and women^44^. Recent safety data from HPTN084 suggest CAB-LA use is safe also in pregnant and lactating women^58^. Since 2022, the WHO has recommended that CAB-LA be offered as an additional prevention choice to those at substantial risk of HIV infection. CAB-LA is licensed for prevention in 53 countries worldwide and is starting to be rolled out in 11 countries in Africa^48^. However, supplies are limited and CAB-LA is not available to all who would choose it. So far, 15,000 people globally—but <3,000 African women—have been started on CAB-LA^48^.

An important implication of CAB-LA scale-up is that infections occurring just before CAB-LA initiation (not detectable at the time of treatment initiation) or while taking CAB-LA are difficult to detect using standard testing algorithms. This is due to altered (clinically silent) presentation of acute HIV infection in people taking CAB-LA, a phenomenon known as ‘long-acting early viral inhibition’. Delayed diagnosis is associated with resistance to certain antiretroviral drugs including dolutegravir, a critical part of first-line HIV therapy regimens in most countries in Africa^59^. Modeling the cost-effectiveness of CAB-LA in SSA while taking account of the risk of drug resistance suggests that CAB-LA has the potential to avert more deaths over 50 years than daily oral PrEP, and would be cost effective if delivered at the same cost as oral PrEP^60^. The cost-effectiveness of CAB-LA has also been studied in South Africa, which has a much higher gross domestic product per capita than most other countries in the region. CAB-LA was not found to be cost effective when targeted to those at substantial risk unless the price could be reduced^60–62^. One model of maximum cost at which CAB-LA would be similarly cost effective to oral PrEP (in South Africa) found a per-dose cost ranging from $9.05 to $14.47, which is considerably less than current costs. The extent to which models differ in their findings of PrEP cost-effectiveness relate to assumptions about the degree to which it is effectively used by individuals—mostly only during periods in which they are likely to have new condomless partners—which is uncertain^60–62^.

The injectable antiretroviral capsid inhibitor lenacapavir is an even longer-lasting PrEP option, requiring subcutaneous injection only every 6 months. The randomized component of a large phase 3 trial (PURPOSE 1) of lenacapavir in African women was stopped in mid-2024 following a preplanned interim analysis, because there were no incident HIV infections in the lenacapavir arm (follow-up of participants continues)—while incidence in two different daily oral PrEP arms was similar to background incidence^63^. This has generated excitement about the possibility of a highly effective, twice-yearly prevention technology soon becoming available. The randomized component of a second trial of lenacapavir for HIV prevention in cisgender men and trans women (PURPOSE 2) was also stopped in late 2024 again for preventing incident infections, and other trials in the USA (https://www.purposestudies.com/) are due to report in 2025, evaluating len-acapavir in women (PURPOSE 3) and injecting drug users (PURPOSE 4). One modeling study assessed the cost-effectiveness of lenacapavir for HIV prevention among individuals at substantial risk in western Kenya, Zimbabwe and South Africa^64^. The maximum price at which lenacapavir could be cost effective varied by setting and by the extent to which eligibility was extended beyond those at highest risk. Wider coverage is predicted to avert more infections but lenacapavir would have to be delivered at even lower costs to remain cost effective; lenacapavir rollout to 1.6–4.0% of the population would avert 12.3–18.0% of infections and could be implemented cost-effectively at a price per dose of $106.30 (South Africa), $21.10 (Zimbabwe) and $16.60 (western Kenya). The potential cost per dose of lenacapavir in low- and middle-income countries is not yet known, but below $40 is thought to be achievable. Researchers projecting the minimum lenacapavir pricing based on generic mass production and a Cost-Plus model found it could be manufactured in bulk for <US$100 per person per year^65^.

There is some disquiet that, without coordinated rollout of CAB-LA and lenacapavir (as did happen for dolutegravir, the preferred first-line antiretroviral treatment) widespread scale-up will likely be delayed^66^. The Coalition to Accelerate Access to Long-Acting PrEP (https://avac.org/resource/coalition-to-accelerate-access-to-long-acting-prep/), which includes the WHO, Global Fund, the US President’s Emergency Plan For AIDS Relief (PEPFAR) and other donors/advocates, has developed a plan for accelerated introduction of CAB-LA—but this plan retains the developer (ViiV Healthcare) as sole supplier in the initial period. This is a lost opportunity that advocacy groups are working to address^67,68^. The developer of lenacapavir (Gilead Sciences) has issued licenses to six generic manufacturers ahead of regulatory approval but this has also led to concerns about access and affordability^69^.

### PEP

HIV PEP as recommended by the WHO is a 28-day course of antiretroviral medication, consisting of tenofovir disoproxil–lamivudine–dolutegravir (TLD), taken within 72 h after potential exposure to HIV^70,71^. PEP works by halting viral replication and preventing persistent infection. There have been no randomized controlled trials assessing the efficacy of HIV PEP. Nonhuman primate studies suggest that PEP may reduce the risk of acquiring HIV by around 90%^72^, with the efficacy likely to be higher the earlier PEP is initiated after sexual exposure. Based on studies in macaques, even a single dose of prophylaxis with tenofovir alafenamide (TAF)/emtricitabine (FTC) and an integrase inhibitor (elvitegravir) before or after sex leads to over 90% protection, leading the authors to conclude it is a promising HIV prevention strategy^71^. Likewise, Bekerman et al.^73^ found that two doses of TAF/FTC plus the integrase inhibitor bictegravir initiated within 24 h of exposure provided over 80% protection, greater than the protection offered with TAF/FTC alone. These studies suggest that HIV PEP can reduce the risk of infection if taken quickly after exposure and for a long enough period. Animal studies suggest that 28 days of PEP may not be required to prevent infection; if PEP is started rapidly (for example, within a day at most), a much shorter course would likely be effective, although there are no data in humans to confirm this. Also of note, PEP has the potential to act as a ‘gateway’ to PrEP initiation^74^.

TLD is the first-line therapy for millions of people living with HIV across Africa and thus is widely available and considered safe. Although PEP has been recommended by the WHO for years, in practice it is not easily accessible. It requires a prescription and, in many settings, it is reserved for people who have experienced sexual assault or an occupational exposure. The WHO have recently revised their guidelines to emphasize the importance of expanding access to PEP to all who are potentially exposed to HIV, making it easily available in communities so it can be initiated rapidly after exposure^70^. Monitoring PEP use with HIV self-testing was also recommended^47^. Although there are no randomized controlled trials to confirm the impacts of this approach, its potential benefits are considered to outweigh potential harms.

## Financing biomedical prevention

The scale of the challenge posed by HIV to African health systems in the 2000s and 2010s was unprecedented, with life expectancies in many countries falling (for example, to age 45 in Malawi and 33 in Zimbabwe in 2002) and wider economies buckling under the strain of the epidemic. The global community coalesced to generate funding to address this challenge. In 2002, the Global Fund to Fight for AIDS, Tuberculosis and Malaria (GFATM; https://www.theglobalfund.org/en/) was established and, in 2003, PEPFAR (https://www.state.gov/pepfar/) was launched. Today, both institutions account for almost all HIV funding in low-income African countries with high HIV prevalence (for example, in Malawi, Zimbabwe and Mozambique), whereas domestic funding for HIV has increased substantially only in relatively wealthier countries (for example, South Africa and Botswana).

The global response to HIV has been an outstanding success, as evidenced by the fall in HIV infections, but there are concerns that the exceptional levels of international funding for HIV may not be sustained into the future. This is a challenging issue, as global priorities shift toward new concerns such as climate change and pandemic preparedness. International funding for HIV has flatlined in recent years^1,75^, and it is widely expected to fall in future. There are hopes that the fall can be mitigated by increased domestic funding for HIV, but this will not replace donor funding in full because countries face a myriad of other health, social and economic challenges. If funding does fall, this is likely to have adverse implications for HIV prevention, unless domestic funding can fill the gap.

Several countries have experimented with innovative financing mechanisms, such as the use of taxes/levies and debt conversion instruments, to increase the availability of domestic funds for HIV services. The Zimbabwe National AIDS Trust was established in 2000 and receives revenues from a tax on formal sector employers and employees, with 10% of proceeds going to prevention^76^. Cote d’Ivoire and Cameroon have recently agreed schemes with creditors, through the GFATM’s Debt4Health Swap, for their debt burdens to be reduced with released funds being committed to national HIV responses^77^. Given the moral imperative for countries to provide antiretroviral therapy to citizens living with HIV for their rest of their lives, it has been recognized that HIV infections create a financial quasi-liability which is comparable in some African countries to management of their ratio of debt to gross domestic product^78^. In such a context, it makes sense for countries to target available funds to maximally reduce new HIV infections, especially among women and those at highest risk. To date, many of the most prominent HIV prevention programs that focus on women have been financed primarily using international funding.

There are risks that future financing of HIV prevention services will, therefore, be inadequate unless domestic contributions substantially increase. This requires a combination of science and advocacy. Encouragingly, health leaders across Africa appear to recognize the challenge. For instance, African heads of state committed in 2019 to the African Leaders Meeting Investing in Health Declaration, aiming to increase domestic spending on health, especially to meet the challenges posed by HIV^79^. It is critical that HIV prevention continues to be bolstered, to both ease fiscal challenges and ensure population health improvement.

## Accelerating effective coverage of biomedical prevention tools

As described above, we have highly effective tools for preventing acquisition of HIV (all of which have been shown to be safe) and many of these should, by now, be well embedded in health systems. In reality, however, too few people are accessing prevention tools effectively. Randomized trials in East Africa have shown that offering individuals the choice of whichever biomedical prevention method best fits their needs (oral PrEP, long-acting injectable PrEP or condoms and PEP in the event of exposure) and the option to change their choice if their circumstances or preferences change, increases coverage of high-risk transmission events and results in substantially more people initiating primary prevention^80,81^.

The challenge for new technologies, which include new drug delivery mechanisms, is that they be developed in ways that will reduce barriers to their ongoing use, such as by ensuring privacy and ease of use. Many barriers can be mitigated without new technologies, by using the ones we already have better. For example, many countries have been reluctant to adopt WHO’s recommendation to de-medicalize and monitor PrEP or PEP use via HIV self-tests. Similarly, task shifting of PrEP/PEP prescribing to community cadre, pharmacies and/or self-care has not been adequate. There is resistance among stakeholders to changing existing prescribing, delivery and monitoring practices and regulations. Although such regulations are in place to ensure population safety, they are often maintained by vested interests (for example, health-care professionals’ concerns about ‘losing control’ over certain aspects of care)^82^.

One way to make PEP more widely available is to provide TLD (WHO-recommended PEP) in communities for anyone to access without prescription. This approach would need to be coupled with community education to promote PEP following condomless sex. Freely available TLD might end up being used either as PrEP by people wanting to prevent infection or as treatment by people living with HIV, allowing them to circumvent the need to go to a clinic (even though these would not be the intended uses). Phillips et al.^83^ modeled effectiveness and cost-effectiveness of this approach; assuming a high uptake of TLD, they projected a mean reduction in general population HIV incidence in eastern and southern Africa of 31% over 20 years. Nonprescription, community-based TLD was cost effective in 90% of scenarios and cost-saving (in terms of disability-adjusted life years) in 58% of scenarios. A range of implementation research projects are currently underway to make PEP more easily available including through pharmacies, vouchers, community cadres and ‘PEP in Pocket’, but none have yet tested removing all barriers to TLD access^84,85^.

Multipurpose prevention technologies combining antiretrovirals and contraception are a promising intervention for women who want to prevent HIV and pregnancy simultaneously^86^. Although reported acceptability and adherence to a dual PrEP/contraceptive pill was high among adolescent girls and young women in Zimbabwe, mean adherence was too low for protection, which did not differ between the dual PrEP/contraceptive pill and taking two pills separately^87^. Ongoing studies investigating a co-formulated smaller pill may give a better picture of adherence and impact^88^. A 3-month combined dapivirine–levonorgestrel vaginal ring was well tolerated, with plasma and cervicovaginal levels that compared well with a dapivirine-only ring and other levonorgestrel contraceptives^89^. Other future approaches under development include a subdermal implant that can be refilled by transcutaneous injection for ultra-long-acting delivery of antiretrovirals for PrEP, as well as long-acting oral prevention^90^.

A major challenge is to identify and test the population impact of scalable implementation strategies, to ensure effective use of available technologies while advocating for coordinated introduction of new innovations (see Box 3 for implementation priorities). Proactively engaging communities of women will increase understanding that HIV is genuinely preventable and worth preventing. In Mysore, India, for example, within the Ashodaya Samithi sex worker collective, sex workers designed and implemented PrEP rollout as an integral part of their broader sexual health and social support program. Importantly, sex workers living with HIV were at the heart of community mobilization for PrEP, and were able to discuss the value of remaining uninfected with sex workers who were HIV-negative. In stark contrast to the numerous other demonstration programs run over that period, over 90% of all potential PrEP users opted to initiate PrEP; 86% of those were retained after 16 months, of whom 87% had protective levels of PrEP^91,92^.

Separately, there has been concern about ‘risk compensation’ among those using biomedical prevention, whereby condom use is reduced and rates of STIs rise. Evidence for this is limited^93^ as levels of condomless sex and STIs are already high in Africa and the high levels of efficacy for HIV prevention outweigh the potential behavioral impact. PrEP services are an opportunity to provide sexual health promotion, STI testing and treatment to those at highest risk of HIV and STI acquisition.

## Future research priorities

The immediate challenge for prevention research is to determine how to deploy the highly effective prevention tools already available (or nearly available) to ensure effective coverage among women who are at a substantial risk of infection. Evaluation of new biomedical tools including preventive vaccines will of course be required in the longer term if the goal of eradicating HIV infection is to become a reality. To maximize the impact of available and emerging technologies in the near future, equal priority must be given to efficacy and implementation research. Below, we outline key considerations for research in both of these areas.

## Determining the efficacy of biomedical prevention products

While the falling global incidence of HIV and growing availability of effective primary prevention tools is a cause for celebration, it has implications for research aimed at assessing the efficacy of new biomedical prevention tools. Falling incidence rates mean that trials need to recruit more people to demonstrate impact, and they must be large enough to detect benefit or equivalence compared to existing products, rather than placebo. An additional complication is that the effectiveness of oral PrEP—a likely comparator to new innovations—is highly dependent on adherence, making it difficult to estimate efficacy. All of this poses challenges to the use of traditional randomized clinical trials that require impractically large sample sizes and non-inferiority designs. A consensus group of academic researchers, regulators, pharmaceutical innovators and other stakeholders (the Forum HIV Prevention Trial Design Project) proposed estimating HIV incidence in people not on PrEP as an external counterfactual, measured using a recent infection testing algorithm—to which on-PrEP incidence in trial participants could then be compared^94^.

This approach was adopted by the recent PURPOSE 1 trial^63^, which tested the effectiveness of 6-monthly injectable lenacapavir and TAF by comparing prospectively measured HIV incidence for each investigational agent to background HIV incidence among all those screened to take part in the trial (a cross-sectional incidence cohort). HIV incidence in each drug arm was also compared to that in an active internal control group receiving daily oral TDF. This new approach not only demonstrated the efficacy of lenacapavir, but also confirmed the difficulty women have in taking daily oral PrEP (incidence in the oral PrEP arm matched that in the corss-sectional incidence cohort). Therefore, it is likely that this approach will be used to assess emerging antiretroviral-based products.

A similar approach could potentially be used to assess the efficacy of new vaccines or broadly neutralizing antibodies, although it is more likely that these trials will use a placebo arm but allow access to standard-of-care PrEP in all trial arms, for anyone who chooses it. Additionally, investigators might combine trial resources to determine efficacy of different agents simultaneously—as happened in the PrEPVacc study, which combined trials of HIV vaccines with a trial of a novel oral PrEP agent (TAF) in women^95^.

### Implementation research to develop and test models of prevention delivery

Implementation research is critical to scaling up efficacious prevention approaches in a way that provides effective coverage for those at risk of HIV acquisition. Having an effective prevention product is not in itself a guarantee of uptake. There is growing consensus that prevention needs to be de-medicalized; people who use it are, after all, not ill. Thus, research on how best to remove barriers to access and use (Box 2) can help identify changes required at different levels of health programming. Furthermore, implementation research can investigate how advances in diagnostics and digital technologies can support shifting prevention delivery to self-care or community cadre, safely and integrated within health programming^96–99^.

Box 4 summarizes priorities for future HIV prevention research that can scale up effective technologies among African women. Communities should be at the center of prioritizing, designing, implementing and evaluating interventions. Citizen science can play an important role in determining why and how deployment is failing or succeeding, as well as helping tailor deployment strategies to specific settings and populations^100^.

## Conclusion

Accelerating prevention of new HIV infections among African women and their sexual partners is a global health priority. Recent declines in HIV incidence have been driven by both treatment expansion and combination prevention, underpinned by a growing toolbox of prevention commodities combined with strong understanding of patterns of risk, and how to reach those with the greatest need.

The next phase of the response will be driven by three key pillars. First, is to strengthen the implementation of HIV prevention. Implementation research will be needed to drive innovation in developing community leadership models, strengthening delivery platforms, expanding choice (both of products and delivery platforms) and tailoring strategies to address gaps. Second, is to develop and test new products and integrate them within the prevention toolbox. Ongoing research on new longer-acting formulations of antiretrovirals, candidate vaccines and other approaches such as broadly neutralizing antibodies will need innovative methodologies to adapt to the new prevention landscape. Third, is work to both reduce the prices of emerging products and to ensure long-term financing of the response. This must be underpinned by country-specific priorities, cofinancing, modeling and cost-effectiveness research to drive decision-making.

## Box 1 The HIV prevention cascade

The HIV prevention cascade offers a framework to capture the steps needed to achieve optimal effective coverage with prevention products, in this case antiretroviral-based primary prevention products, among women in Africa. The prevention cascade is more complex than the treatment cascade for a range of reasons; for example, there are many prevention options and the risk of infection (and, therefore, need for preventive tools) is highly variable between place, person and time. Consequently, many different cascades have been proposed and there is no single agreed model—and it is unlikely there will be one in the future. One proposed ‘unifying’ approach to the prevention cascade has quite strong overlaps with the COM-B model of behavior, which outlines capability, opportunity and motivation as the three crucial determinants of behavior change. In the context of HIV prevention, these can be described as motivation to adopt the prevention tool (pillar 1), access to any particular commodities required (pillar 2) and capabilities for effective use (pillar 3). It identifies key reasons between gaps in the cascade, which include, but are not limited to, insufficient knowledge, risk perception or norms for motivation; limited availability, affordability or acceptable provision for access; and limited capabilities, self-efficacy or skills for effective use.

## Box 2 Barriers to uptake and continuation of PrEP/PEP

Barriers are listed below, grouped according to three prevention cascade pillars—motivation and intention, access and capability

**Cascade pillar 1: Motivation and intention to use PrEP and PEP**

- Low perception of risk
- Low awareness about PrEP and its potential to prevent HIV acquisition; even lower awareness of PEP
- Prevention is a low priority compared to other challenges of daily life—and often for those at highest risk, fatalism around HIV acquisition
- Perception of PrEP use as stigmatizing
- Perceived or experienced side effects
- Skepticism about effectiveness
- Distrust between sexual partners

**Cascade pillar 2: Access to PEP and PrEP**

- Rapid scale-up of facilities providing PrEP in some countries but very limited or no access in many settings
- Limited integration of PrEP into sexual reproductive health and antenatal services
- Universally poor access to PEP
- Personal and social barriers to accessing available health services (including distance, waiting times, stigma and fear of being recognized or treated poorly at health facilities)

**Cascade pillar 3: Capabilities for effective use**

- Inconvenience of carrying pills and returning for refills
- Fear that taking PrEP might be misconstrued as taking antiretroviral therapy or might inadvertently expose the persons’ sexual activity to family and friends
- Lack of clear guidance for both providers and clients on how to approach discontinuation and reinitiation of PrEP

## Box 3 Priorities for implementation of existing HIV prevention technologies

**Cascade pillar 1: Motivation/intention to use PrEP and PEP**

- Facilitate community-led education and empowerment to reduce stigma
- Increase awareness of sexual rights and improve knowledge and self-efficacy of prevention technologies and where and how to access them

**Cascade pillar 2: Access to PEP and PrEP**

- Coordinate introduction of injectable PrEP with the aim of lowering costs and increasing volumes available
- Ensure PEP is easily and widely available throughout communities and that people are empowered to use it
- Facilitate and promote self-care in communities through access to self-testing and community delivery of interventions, thereby lowering the barriers to access for all PrEP technologies
- Maximize the choice of prevention technologies currently available

**Across the cascade continuum**

- Address the following—regarding motivation, intention, access and capability—as required for each setting and population group:
- Improve quality and effective coverage of STI management
- Intensify and strengthen programs targeting key populations, particularly sex workers
- Ensure adolescents have the motivation, access and capability to use effective and comprehensive sexual and reproductive health services
- Improve effective treatment coverage among men, particularly mobile men and those who are hard to reach (including men who pay for sex)

## Box 4 Future research priorities

1. Find ways to de-medicalize HIV prevention—such as ‘task-shifting’ provision of preventive technologies to community members and moving it away from clinics.
2. Develop capacity for citizen science initiatives focused on HIV prevention and community-led monitoring of services and policies.
3. Evaluate models for safety, acceptability and effectiveness of ‘PEP in Pocket’ that is, providing PEP before exposure for individuals at high risk of acquiring HIV.
4. Evaluate use of community-wide, freely available TLD to lower access barriers for PEP, potentially incorporating a test of 7-day PEP.
5. Determine the role of social media, electronic clinical management systems, digital diagnostics and other digital technologies (including AI-enabled toolkits) to support self-management and community cadre supported care.
6. Determine the role of sexual and reproductive services (including for STIs) and maternal and child health services as a hook to attract sexually active populations into prevention services— where risk stratification can target products to those who need them most.
7. Evaluate the role of community-based cadres in tailoring prevention packages to individual needs and the extent to which this contextual knowledge is adaptive to changing epidemiology.
8. Identify levers to support intrinsic motivation (perceived self-benefit) and extrinsic motivation (external reward or benefit) for effective use and how or whether it is cost effective to deploy these.
9. Determine the impact of biomedical HIV prevention on STIs and their treatment, and any knock-on impact on antimicrobial resistance of *Neisseria gonorrhoeae* in Africa.
10. Explore how HIV prevention activities can be delivered within limited health system capacities, cost-effectively and equitably.
11. Determine the optimal mix of funding sources for HIV prevention, including for populations/interventions seen as lower priority for some governments.
12. Collaborate with policymakers to understand the evidence requirements to lower barriers to prevention technology access.
